# Notoginsenoside R1 Protects Against High Glucose-Induced Cell Injury Through AMPK/Nrf2 and Downstream HO-1 Signaling

**DOI:** 10.3389/fcell.2021.791643

**Published:** 2021-12-01

**Authors:** Fawang Du, Huiling Huang, Yalin Cao, Yan Ran, Qiang Wu, Baolin Chen

**Affiliations:** ^1^ Department of Cardiology, Guizhou Provincial People’s Hospital, Guiyang, China; ^2^ Department of Cardiology, The First Affiliated Hospital of Sun Yat‐Sen University, Guangzhou, China; ^3^ Department of Nephrology, Guizhou Provincial People’s Hospital, Guiyang, China; ^4^ Nanmingtang Clinic, Guizhou Provincial People’s Hospital, Guiyang, China

**Keywords:** notoginsenoside R1, high glucose-induced cell injury, AMPK, Nrf2, apoptosis, diabetic cardiomyopathy

## Abstract

Notoginsenoside R1 (NGR1), the primary bioactive compound found in Panax notoginseng, is believed to have antihypertrophic and antiapoptotic properties, and has long been used to prevent and treat cardiovascular diseases. However, its potential role in prevention of diabetic cardiomyopathy remains unclear. The present study aimed to investigate the mechanism of NGR1 action in high glucose-induced cell injury. H9c2 cardiomyocytes were cultured in a high-glucose medium as an *in-vitro* model, and apoptotic cells were visualized using TUNEL staining. Expression of Nrf2 and HO-1 was measured using Western blotting or reverse transcription-quantitative PCR (RT-qPCR). The Nrf2 small interfering (si) RNA was transfected into cardiomyocytes using Opti-MEM containing Lipofectamine^®^ RNAiMAX. NGR1 protected H9c2 cardiomyocytes from cell death, apoptosis and hypertrophy induced by high glucose concentration. Expression of auricular natriuretic peptide and brain natriuretic peptide was remarkably reduced in NGR1-treated H9C2 cells. Western blot analysis showed that high glucose concentration markedly inhibited AMPK, Nrf2 and HO-1, and this could be reversed by NGR1 treatment. However, the cardioprotective effect of NGR1 was attenuated by compound C, which reverses Nrf2 and HO-1 expression levels, suggesting that AMPK upregulates Nrf2 and HO-1 gene expression, protein synthesis and secretion. Transfection of H9C2 cells with Nrf2 siRNA markedly reduced the cardioprotective effect of NGR1 via reduced expression of HO-1. These results indicated that NGR1 attenuated high glucose-induced cell injury via AMPK/Nrf2 signaling and its downstream target, the HO-1 pathway. We conclude that the cardioprotective effects of NGR1 result from upregulation of AMPK/Nrf2 signaling and HO-1 expression in cardiomyocytes. Our findings suggest that NGR1 treatment might provide a novel therapy for diabetic cardiomyopathy.

## Introduction

Diabetic cardiomyopathy (DCM), a specific form of cardiomyopathy, is characterized by myocardial fibrosis and cardiomyocyte hypertrophy and apoptosis, and is the leading cause of mortality in diabetic patients (Jia et al., 2018). Epidemiological studies have demonstrated that diabetic patients are more likely to develop cardiac abnormalities than patients without diabetes (Zhou et al., 2021). Heart failure is a major complication of diabetes, which has been caused not only by atherosclerotic cardiovascular diseases, but also by DCM. Recently studies had showed that even in patients with prediabetes, the risk of heart failure was increased (Cai et al., 2021). Furthermore, in those patients with established HF, prediabetes was associated with a worse prognosis (Mai et al., 2021). Although substantial advances have been made in DCM research over the past few years, the underlying molecular mechanisms of DCM are not yet fully understood. In addition, there are currently no disease-specific drugs for DCM available in the clinic.

Notoginsenoside R1 (NGR1) is a bioactive saponin belonging to the notoginsenoside family of compounds isolated from the genus *Panax* (ginseng), which possesses antioxidant, anti-inflammatory and antiapoptotic properties (Sun et al., 2013). By activating the Nrf2/HO-1 pathway, NGR1 has neuroprotective effects on cerebral ischemia/reperfusion injury (Meng et al., 2014). Previous research has demonstrated that NGR1 exerts cardioprotective effects on ischemic damage (Yu et al., 2016) and protects against DCM by inhibiting apoptosis and oxidative stress, which ultimately suppresses ventricular fibrosis and hypertrophy (Zhang et al., 2018). These studies have focussed on the potential cardioprotective effects of NGR1.

Consequently, it is important to clarify the molecular mechanisms of DCM and explore compounds that can potentially be used to treat it. Adenosine monophosphate-activated protein kinase (AMPK) is one such promising target, as preliminary evidence points to its cardioprotective role in pathological cardiac hypertrophy. Cardiomyocyte hypertrophy is an adaptive response to hemodynamic overload and cardiomyocyte injury. At the cellular level, cardiac hypertrophy is characterized by an increase in cardiomyocyte size and increased synthesis of various cardiac hypertrophy markers, including auricular natriuretic peptide (ANP) and brain natriuretic peptide (BNP) (Syed et al., 2016). Heme oxygenase-1 (HO-1) is an endogenous cytoprotective enzyme produced in response to electrophilic and oxidative stress; its transcriptional activation of cytoprotectant genes is regulated by the nuclear factor-erythroid 2-related factor2 (Nrf2) (Aleksunes et al., 2010). Recent evidence suggests that Nrf2 is activated by the AMPK pathway, which enhances HO-1 expression and reduces cellular damage (An et al., 2020). These processes offer potential targets for treatment of diabetic cardiomyopathy. However, the pathophysiology of diabetic cardiomyopathy is incompletely understood. Whether NGR1 can ameliorate diabetic cardiomyopathy, and the mechanisms by which it regulates the AMPK/Nrf2/HO-1 signaling pathway, have not yet been determined. Therefore, *in-vitro* experiments were carried out in this study to explore the cardioprotective effects of NGR1 on high glucose-induced injury and whether it acts via upregulation of the AMPK/Nrf2 signaling pathway.

## Materials and Methods

Cell culture and treatments. Rat H9c2 cardiomyocytes were purchased from the Cell Bank of the Chinese Academy of Sciences (Shanghai, China). Cells were cultured in DMEM (Gibco; Thermo Fisher Scientific, Inc.) supplemented with 1% streptomycin-penicillin, 10% FBS and 4 mM l-glutamine (Gibco; Thermo Fisher Scientific, Inc.) in an atmosphere of 5% CO_2_ at 37°C. The cells we used are routinely authenticated and tested for *mycoplasma* contamination. The medium was changed every 3 days, and the cells were subcultured when the density reached 80% confluence. Samples were then placed into a single cell suspension, after which 1 × 10^5^ cells/mL were seeded into 6-well plates. The H9c2 cardiomyocytes were passed at a ratio of 1:3 at 37°C, 5% CO_2_ and 100% humidity in all experiments. Cells were subsequently divided into different groups according to the experimental requirements. To evaluate the effects of NGR1 on diabetic cardiomyopathy, cultured cardiomyocytes were randomized into four experimental groups: i) Low glucose (LG) control, 5.5 mM), ii) high glucose (HG), where cells were treated with 30 mM glucose (Beijing Solarbio Science and Technology Co., Ltd.) for 24 h; iii) LG + NGR1, and iv) HG + NGR1, where cells were treated with 20 µM NGR1 (Shanghai Aladin Biochemical Technology Co. LTD.) for 24 h. To determine the role of AMPK/Nrf2 signaling in NGR1 cardioprotective action, cells were randomly divided into the following eight experimental groups: i) LG (control); ii) HG; iii) LG + NGR1; iv) HG + NGR1; v) HG + NGR1 + compound C; vi) HG + NC-siRNA; vii) HG + Nrf2-siRNA; and viii) HG + NGR1 + Nrf2-siRNA.

Cell viability and morphological analysis. Cell viability was evaluated using MTT assays and cellular morphological methods. The cells were cultured in 96-well plates (5×10^3^ cells/well) and incubated with a solution of MTT (final concentration of 10 μL/ml) at 37°C for 4 h after the various treatments. Formazan crystals were dissolved in dimethyl sulfoxide (DMSO, 150 μL/well). Cell viability was expressed as a percentage of MTT reduction. Morphological changes were observed by light microscopy (×100 magnification, OLYMPUS, Japan).

Terminal Deoxynucleotidyl Transferase-mediated dUTP Nick End Labelling (TUNEL) Staining. TUNEL assays were performed using the TUNEL Apoptosis Detection kit (Roche Applied Science, Mannheim, Germany) according to the manufacturer’s instructions. Cells were fixed with 4% paraformaldehyde for 15 min. After three successive washings in PBS, the cells were permeabilized with 0.1% Triton X 100 (Biyuntian Biological Co., Ltd.) for 10 min. Sections were exposed to recombinant TdT enzyme and Alexa Fluor 647–12-dUTP labeling mix for 60 min at 37°C. Images were captured with a fluorescence microscope. After washing with PBS, DAPI (Biyuntian Biological Co., Ltd.) was used to stain the nuclei of normal cells blue, while apoptotic cells were stained red. The TUNEL-positive cells were quantified from three random fields at a magnification of ×400.

Western blotting. H9C2 cardiomyocytes were lysed in RIPA buffer using a mixture of protease inhibitors (Biyuntian Biological Co., Ltd.). Total protein content in each sample and marker was determined using a BCA protein assay kit (Biyuntian Biological Co., Ltd.). Each protein sample (40 μg) was diluted 10 times, after which standard protein dilutions were prepared in duplicate at concentrations of 1, 0.8, 0.6, 0.4, and 0.2 mg/ml. Polyvinylidene difluoride (PVDF) membranes were soaked with methanol and placed in transfer buffer together with filter paper. The positive pole of the membrane transfer instrument was positioned in the buffer, and the following were added in order: three layers of filter paper, PVDF membrane, gel, three layers of filter paper, and the negative pole. The protein samples were first run on SDS-PAGE (80 V at 30 min*,* 120 V at 120 min), and then transferred to a PVDF membrane (Millipore, Darmstadt, Germany) for 50–120 min at 200 mA (ANP and BNP, 200 mA for 50 min; β-actin and HO-1, 200 mA for 90 min; Nrf2, 200 mA for 120 min). Antibodies used for western blotting were as follows: ANP (1:1,000; Affinity Bioreagant, catalogue number DF6497), Nrf2 (1:1,000; Wuhan Sanying Biotechnology, catalogue number 16396-1-AP), HO-1 (1:2000; Wuhan Sanying Biotechnology, catalogue number 10701-1-AP), *p-*AMPK (1:1,000; CST, Ser485, 2,537),β-actin (1:500; Wuhan Boster Biological Technology Co., catalogue number BM0627) and BNP (1:500; Abcam, catalogue number Ab19645). Antibody labeling was carried out overnight at 4°C. Protein bands were visualized by an enhanced-chemiluminescent (ECL) reagent (Vazyme Biotech) and exposed to X-ray film (Thermo Fisher Scientific, Inc.). Band density was analyzed and quantified using NIH Image software (ImageJ, 1.37v).

Reverse transcription-quantitative PCR (RT-qPCR). RNA was extracted using TRIzol^®^ reagent (Aidlab Biotechnologies Co., Ltd.) The RNA sample (5 µg) was treated with dnase and subsequently used for cDNA synthesis. Real-time PCR was performed on an ABI (QuantStudio 6) instrument using SYBR Green Master Mix (Vazyme Biotech) as follows: 50°C for 2 min, followed by 40 cycles at 95°C for 10 min, at 95°C for 30 s, and 60°C for 30 s. To assess the concentration and purity of total RNA, the optical density (OD) 260:OD 280 (1.8:2.0) ratio of the extracted RNA sample was measured. The concentration of RNA was subsequently calculated according to the OD value using the following formula: Total RNA concentration (µg/µL) = OD260 × 40 × 10^–3^. SYBR Green Master Mix was used for q-PCR with the following primers: Rat β-actin forward, 5′-CAC​GAT​GGA​GGG​GCC​GGA​CTC​ATC-3′ and reverse, (5′-TAA​AGA​CCT​CTA​TGC​CAA​CAC​AGT-3′); rat HO-1 forward, 5′-GCA​TGT​CCC​AGG​ATT​TGT​CC-3′ and reverse, 5′-GGT​TCT​GCT​TGT​TTC​GCT​CT-3′; rat Nrf2 forward, 5′-CCC​ATT​GAG​GGC​TGT​GAT-3′ and reverse, 5′-TTG​GCT​GTG​CTT​TAG​GTC-3′; rat ANP forward, 5′-GGA​AGT​CAA​CCC​GTC​TCA-3′ and reverse, 5′-GGG​CTC​CAA​TCC​TGT​CAA​T-3′; rat BNP forward, 5′-AGA​TGA​TTC​TGC​TCC​TGC​TTT-3′ and reverse, 5′-TGA​GCC​ATT​TCC​TCT​GAC​TTT-3′. The reaction yielded 240, 192, 247, 167, and 145 bp fragments for β-actin, HO-1, Nrf2, ANP and BNP, respectively. Gene expression was normalized with the house keeping gene β-actin.

Nrf2 knockdown via RNA interference. Control siRNA and Nrf2 siRNA were purchased from Wuhan Jingtuosi Biotechnology Co., Ltd. and transfected into H9C2 cells with Opti-MEM containing Lipofectamine RNAiMax (Invitrogen). Four hours post-transfection, cells were seeded in freshly treated DMEM medium or assayed for efficacy of Nrf2 knockdown after another 24-h inoculation.

### Statistical Analysis

Data were expressed as mean ± SEM and analyzed using one-way ANOVA followed by the Bonferroni post-test using SPSS 22.0 statistical software (IBM Corp.). *p* < 0.05 was considered to indicate a statistically significant difference.

## Results

1. NGR1 Protected H9c2 Cardiomyocytes From High Glucose-Induced Cell Death

**FIGURE 1:**
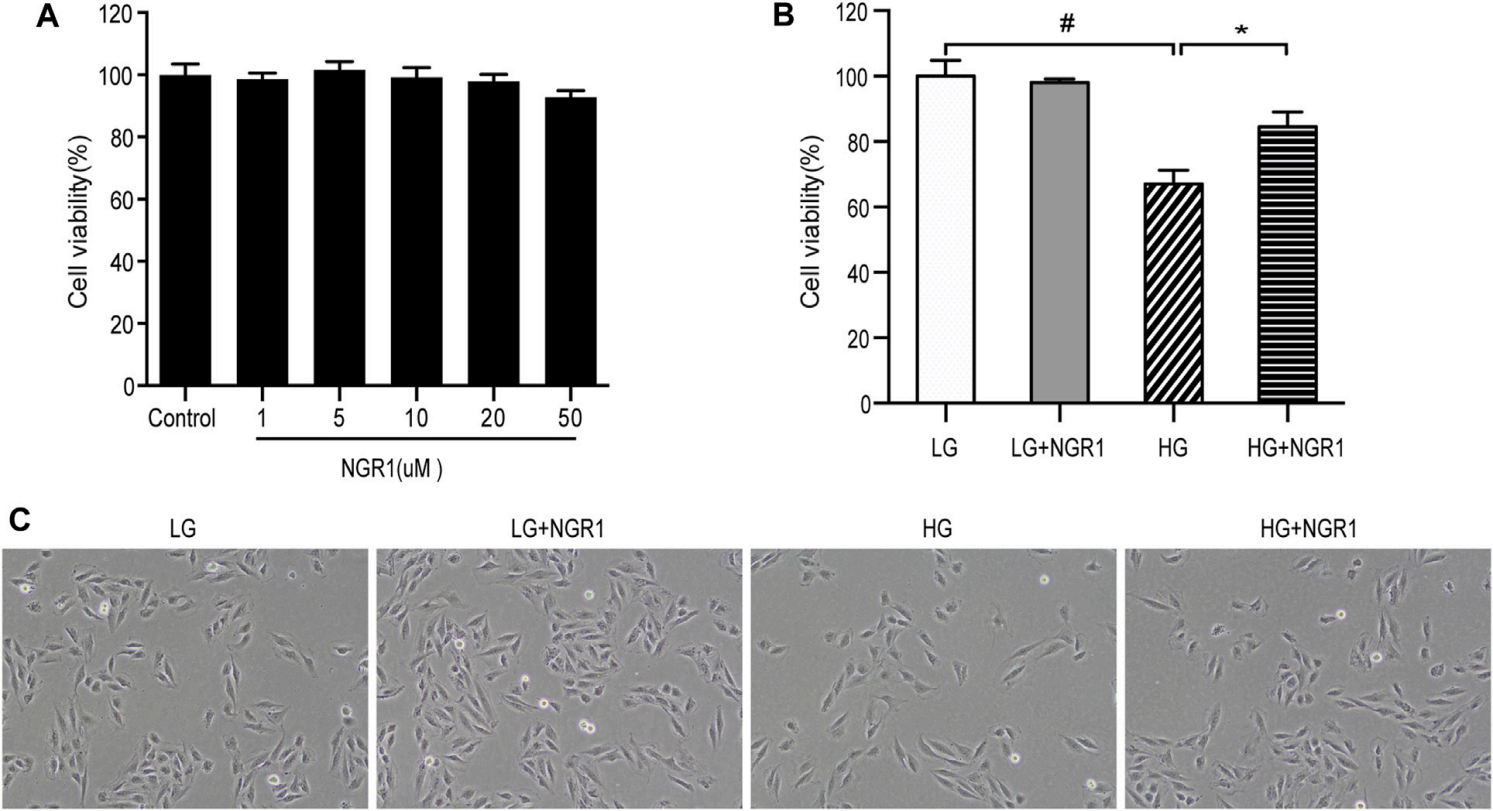
NGR1 protected H9c2 cardiomyocytes from high glucose-induced cell death. **(A)** Effect of NGR1 on H9c2 cardiomyocyte viability, which was insignificant at concentrations up to 50 μM. **(B)** Cell viability decreased when treated with HG, but NGR1 reversed the effect of HG on cell viability. **(C)** Characteristic cell morphological changes at ×100 magnification. The cells swelled and became longer, but these morphological changes were abolished after treatment with NGR1. The data are represented as mean ± SEM, n = 3 for each group. ^*^ *p* < 0.001 and ^#^ *p* < 0.001. LG, 5.5 mM glucose; HG, 30 mM glucose; NGR1, Notoginsenoside R1.

The protective effects of NGR1 on H9c2 cardiomyocyte death induced by high glucose concentration was studied. Firstly, possible cytotoxic effects of NGR1 on H9c2 cardiomyocytes were assessed. Following treatment with different concentrations of NGR1 (1, 5, 10, 20, 50 μM) for 24 h, no significant differences in cell viability were observed (Figure 1A). Secondly, toxicity resulting from high glucose concentration was investigated. The cells were incubated with glucose at different concentrations (0, 30 mM) for 24 h. The MTT assay was then performed to measure cell viability. As shown in Figure 1B, compared with those in the LG control (5.5 mM glucose) group, cell viability in the HG (30 mM glucose) group was reduced to 67.0 ± 1.7% (*p* < 0.001). This study therefore used 30 mM glucose for the HG group in subsequent experiments. Potential cardioprotective effects of NGR1 on H9c2 cardiomyocyte injury induced by high glucose concentration were then examined. NGR1 incubation in the presence of 30 mM glucose (HG + NGR1 group) resulted in cell viability of 84.5 ± 1.84% of the LG control, an increase compared with that of the HG group (67.0 ± 1.70%) (*p* < 0.001) (Figure 1B). In the HG group, cells lost their normal morphology, becoming swollen and elongated, but these morphological changes were abolished in the HG + NGR1 group (*p* < 0.001) (Figure 1C). These results indicated that NGR1 could protect H9c2 cardiomyocytes from high glucose-induced cell death.

2. NGR1 Attenuates H9c2 Cardiomyocyte Apoptosis Induced by High Glucose Concentration

**FIGURE 2:**
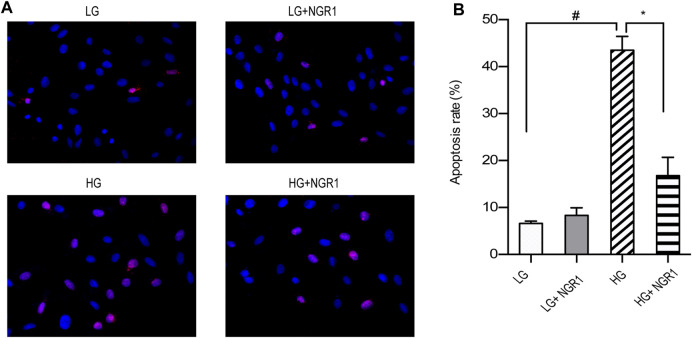
NGR1 attenuates H9c2 cardiomyocyte apoptosis induced by high glucose concentration. **(A)** TUNEL staining at ×400 magnification. High glucose concentration increased H9c2 cardiomyocyte apoptosis, but the effect was reduced by NGR1. **(B)** Pretreatment with NGR1 significantly reduced the apoptosis rate. Data are shown as the means ± SEM of three independent experiments, *p < 0.001 and ^#^ *p* < 0.001. LG, 5.5 mM glucose; HG, 30 mM glucose; NGR1, Notoginsenoside R1.

TUNEL staining was carried out to examine the protective role of NGR1 on high glucose-induced apoptosis. As shown in Figure 2, the apoptosis rate was 43.47 ± 1.71% in the HG group versus 6.60 ± 0.30% in the LG group, and the difference between the two groups was significant (*p* < 0.001). Compared with the HG group, pretreatment with NGR1 reduced the percentage to 16.77 ± 2.51%, which again was significant (*p* < 0.001). These results demonstrate that NGR1 has anti-apoptotic effects on H9c2 cardiomyocytes cultured at high glucose concentration.

3. The AMPK Pathway Mediates The Protective Effects Of NGR1

**FIGURE 3:**
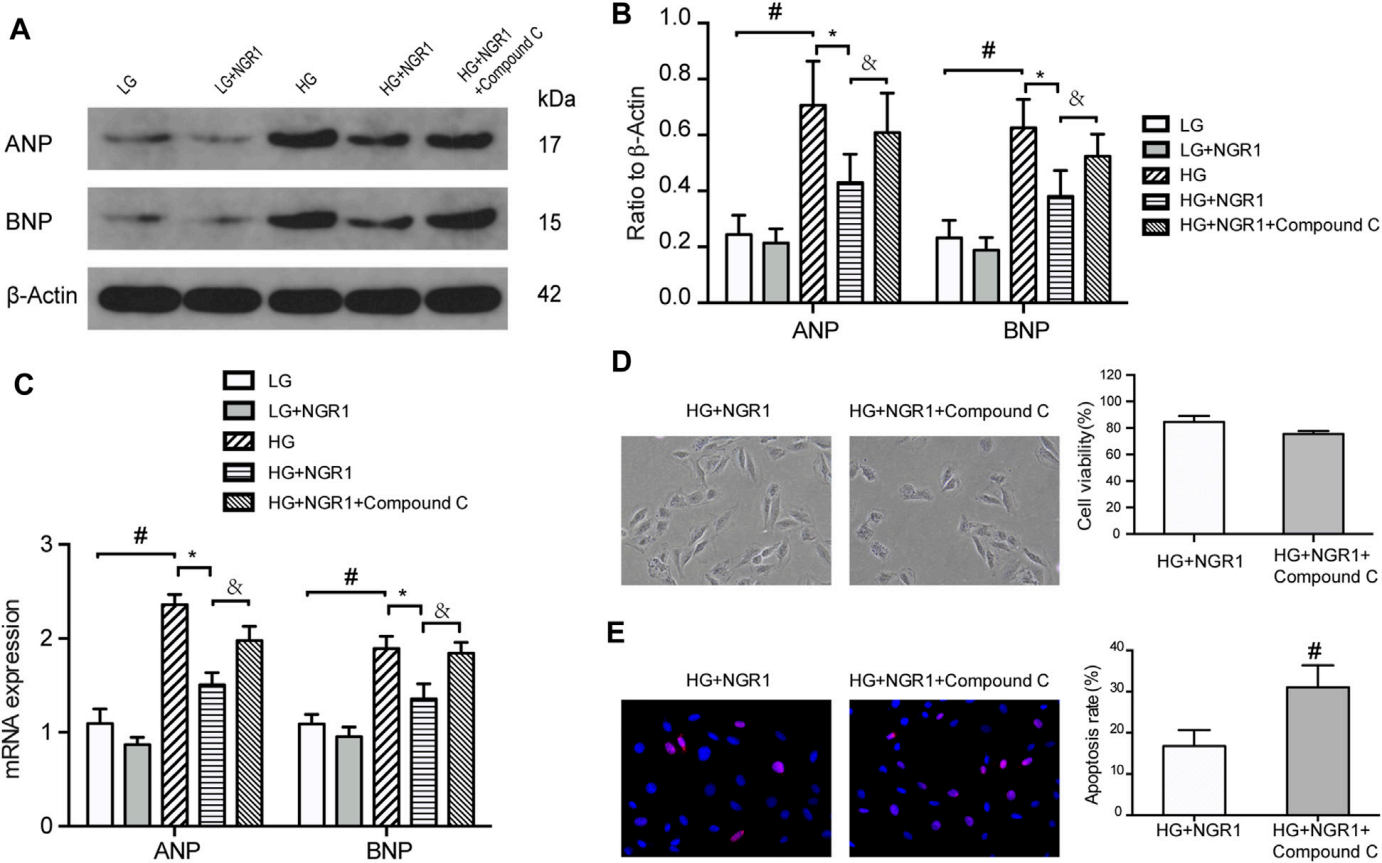
The AMPK pathway mediates the protective effects of NGR1. **(A)** Expression of ANP and BNP proteins in H9c2 cells by Western blot analysis. **(B)** Densitometric analysis of ANP and BNP expression in cardiomyocytes. **(C)** ANP and BNP mRNA expression **(D)** H9c2 cell viability and morphological changes induced by compound C. Cell viability in the compound C-treated group was reduced, and the cells became longer and swollen. Images are at ×200 magnification **(E)** TUNEL staining and apoptosis rate. The apoptosis rate in the compound C-treated group significantly increased. Images are at ×400 magnification. Data are shown as mean ± SEM of three independent experiments, *p < 0.001, ^&^ *p* < 0.001, and ^#^ *p* < 0.001. LG, 5.5 mM glucose; HG, 30 mM glucose; NGR1, Notoginsenoside R1.

Treatment with high glucose significantly increased protein and mRNA expression of ANP and BNP (*p* < 0.001), but these effects were largely reversed by NGR1 treatment (*p* < 0.001) (Figure 3 A–C). These findings are consistent with NGR1 reducing high glucose-induced cardiomyocyte hypertrophy. To determine whether AMPK signaling is involved in the attenuation of high glucose-induced cardiomyocyte hypertrophy mediated by NGR1, compound C was used to block AMPK phosphorylation. Compound C significantly reversed inhibition of cardiomyocyte hypertrophy in response to NGR1 treatment. In addition, compared with the HG + NGR1 group, cell viability in the compound C-treated group was reduced to 75.47 ± 0.85%, and the apoptosis rate increased to 31.04 ± 3.10% (*p* < 0.001). Cells in the compound C-treated group were again swollen and elongated (Figures 3D,E). The above results indicated that compound C reversed the effect of NGR1 on hypertrophy, cell death and apoptosis in high glucose-treated H9c2 cells. Therefore, AMPK is a key factor in the protective effect of NGR1 on glucose-induced cell injury.

4. NGR1 Activates Nrf2 Signaling And HO-1 Expression Via Activation Of The AMPK Pathway.

**FIGURE 4:**
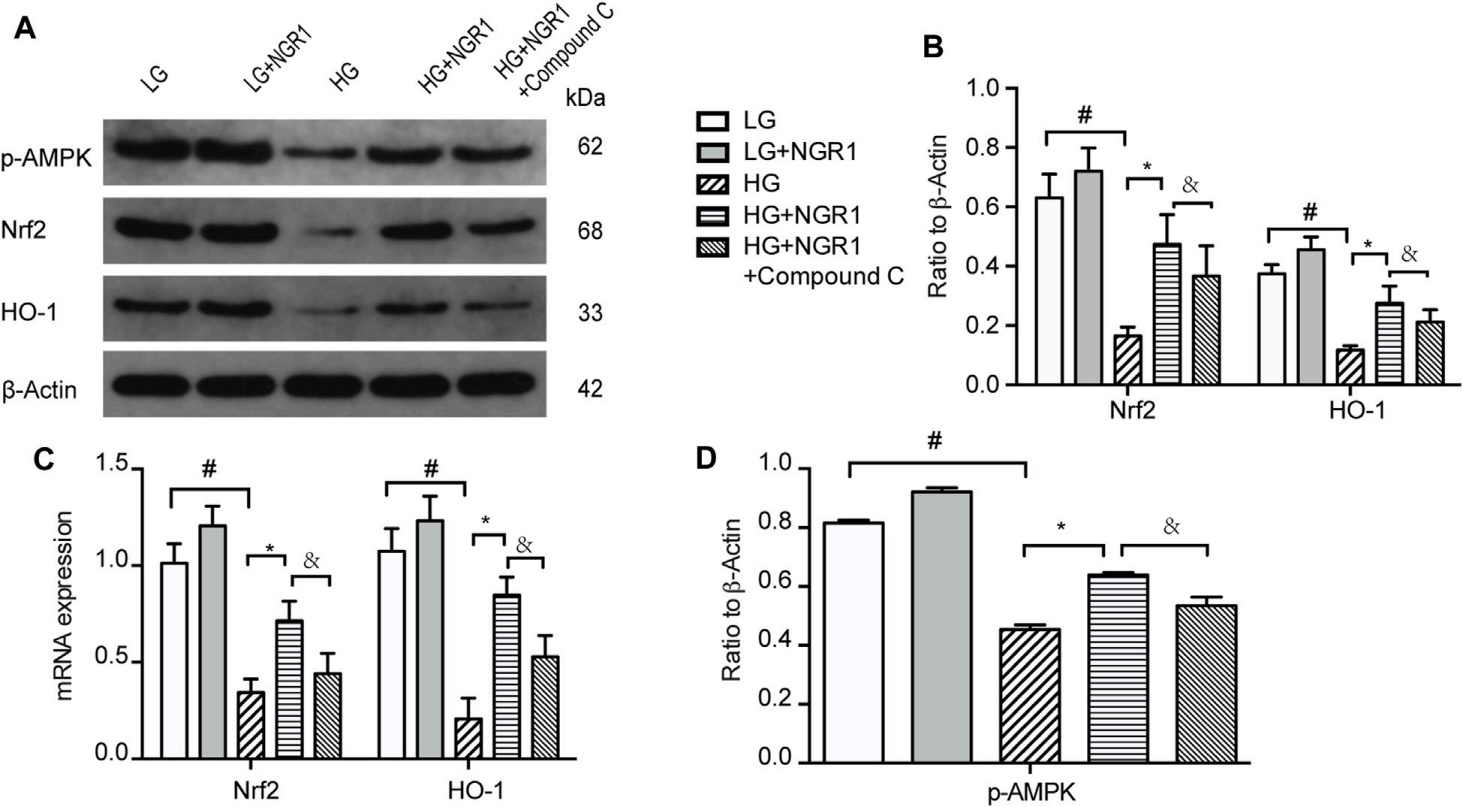
NGR1 activates Nrf2 signaling and HO-1 expression via activation of the AMPK pathway **(A)** Expression of Nrf2 and HO-1 protein in H9c2 cells by Western blot analysis. **(B)** Densitometric analysis of Nrf2 and HO-1 expression. **(C)** Nrf2 and HO-1 mRNA expression **(D)** Expression of *p*-AMPK protein in H9c2 cells by Western blot analysis. Data are shown as the mean ± SEM of three independent experiments, *p < 0.001, ^&^ *p* < 0.001, and ^#^ *p* < 0.001. LG, 5.5 mM glucose; HG, 30 mM glucose; NGR1, Notoginsenoside R1.

To determine whether Nrf2 and HO-1 is involved in AMPK signaling pathway, the impact of high glucose concentration and NGR1 on Nrf2 and HO-1 protein and mRNA expression was assessed. As shown in Figure 4, high glucose concentration significantly inhibited Nrf2 and HO-1 protein and mRNA expression, accompanied by down-regulate the protein levels of *p*-AMPK, and this could be markedly attenuated by pretreatment with NGR1 (*p* < 0.001). These results demonstrated that NGR1 exerts its cardioprotective effect by activating Nrf2 signaling and HO-1 expression. Furthermore, compound C significantly abolished this cardioprotective role by inhibiting Nrf2 and HO-1 protein and mRNA expression (Figure 4). AMPK upregulates gene expression and promotes protein synthesis and secretion of Nrf2 and HO-1. Taken together, these results demonstrate that NGR1 activates Nrf2 signaling and HO-1 expression via activation of the AMPK pathway.

5. NGR1 Exerts Cardioprotective Effects Via Upregulation Of AMPK/Nrf2 Signaling And HO-1 Expression

**FIGURE 5:**
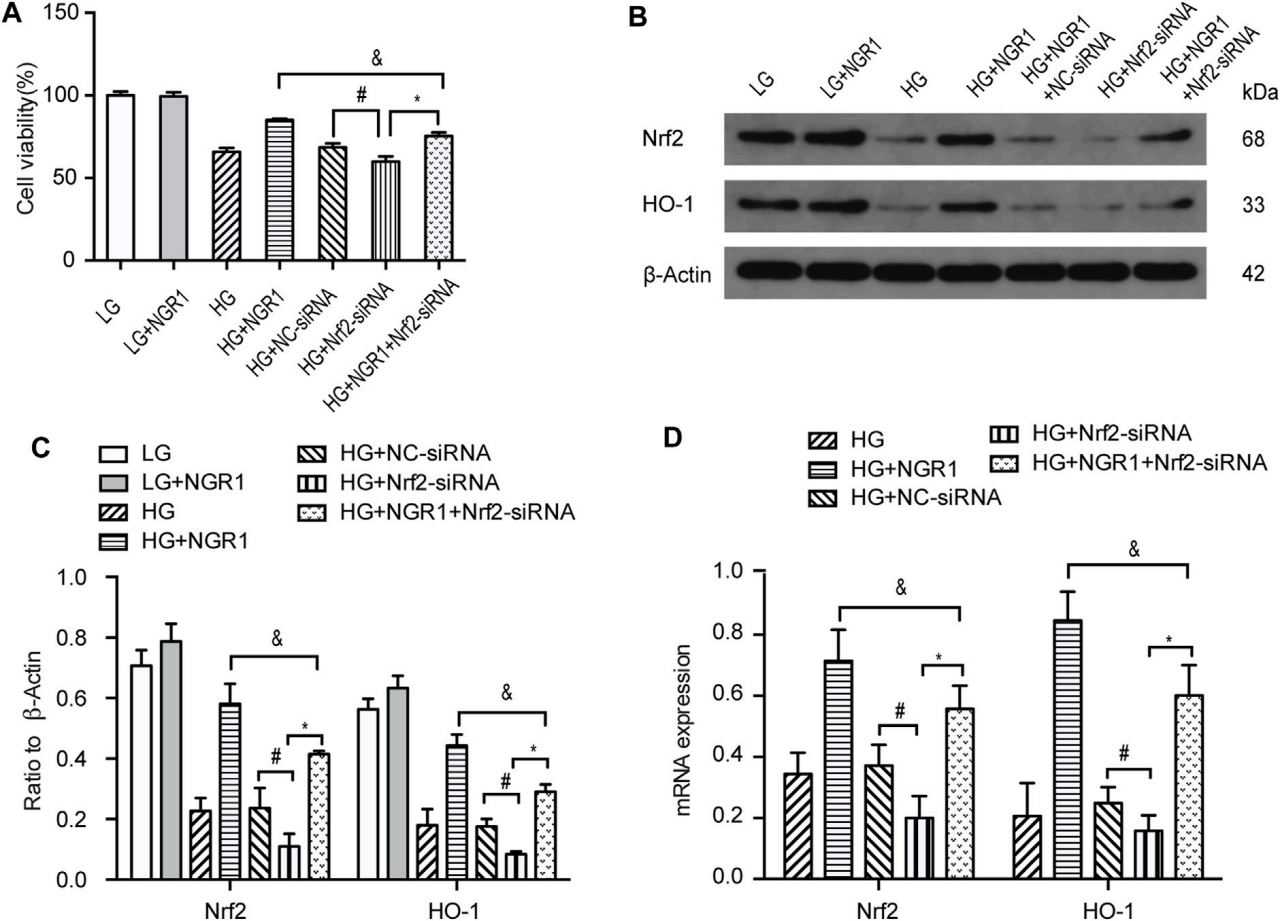
NGR1 exerts its cardioprotective effects via upregulation of AMPK/Nrf2 signaling and HO-1 expression. **(A)** H9c2 cell viability was reduced by Nrf2 siRNA transfection. **(B)** Expression of Nrf2 and HO-1 proteins in H9c2 cells assessed by Western blot analysis **(C)** Densitometric analysis of Nrf2 and HO-1 expression in cardiomyocytes. **(D)** Nrf2 and HO-1 mRNA expression. Data are shown as mean ± SEM of three independent experiments, *p < 0.001, ^&^ *p* < 0.001, and ^#^ *p* < 0.001. LG, 5.5 mM glucose; HG, 30 mM glucose; NGR1, Notoginsenoside R1.

Compared with HG-treated cells transfected with control siRNA (HG + NC-siRNA group), knocking down Nrf2 expression using siRNA (HG + Nrf2-siRNA group) significantly reduced cell viability (68.5 ± 0.99% vs 59.4 ± 1.3%, respectively (*p* < 0.001). However, cell viability was significantly higher in the HG + NGR1 + Nrf2-siRNA group compared with that in the HG + Nrf2-siRNA group, 75.47 ± 0.85% vs 59.4 ± 1.3%, respectively (*p* < 0.001) (Figure 5A). Following NGR1 treatment, expression of Nrf2 and HO-1 was also significantly higher in the HG + NGR1 + Nrf2-siRNA group compared with that in the HG + Nrf2-siRNA group, but lower than in the HG + NGR1 group (*p* < 0.001) (Figures 5B–D). Transfection with Nrf2 siRNA resulted in a significantly reduced cardioprotective effect via decreased expression of HO-1 (*p* < 0.001) (Figures 5A–D). These results show that the cardioprotective effects of NGR1 result from upregulation of AMPK/Nrf2 signaling and HO-1 expression in cardiomyocytes.

## Discussion

In this study we evaluated several effects of NGR1 on cardiomyocytes and the underlying mechanisms by which it functions. The results revealed that high glucose-induced H9c2 cardiomyocyte injury, characterized by cell death, apoptosis and hypertrophy, could effectively be attenuated by NGR1. NGR1 attenuated high glucose-induced cell injury by upregulating AMPK/Nrf2 signaling and expression of its downstream target, the HO-1 pathway. Our findings suggest that NGR1 treatment might provide a novel therapy for diabetic cardiomyopathy.

In recent decades, great strides have been made in understanding diabetic cardiomyopathy. Studies have shown that cardiomyocyte apoptosis and hypertrophy increase in diabetic patients and animals and are correlated with the severity of diabetic cardiomyopathy (Younce et al., 2010; Vulesevic et al., 2016). High glucose concentration is known to cause cardiomyocyte apoptosis and hypertrophy and to play a key role in the pathophysiological development of diabetic cardiomyopathy. Based on these observations, we chose high glucose treatment to model diabetic cardiomyopathy in H9c2 cells. In H9c2 cells, HG (30 mM glucose) treatment reduced viability, promoted apoptosis, and greatly increased ANP and BNP protein and mRNA expression, which in turn inhibited Nrf2 and HO-1 protein and mRNA expression. Consistent with our findings, it has previously been shown that high glucose concentration can suppress Nrf2 activity and subsequent HO-1 expression (Qu et al., 2011)*.* Cardiomyocyte apoptosis induced by high glucose concentration can lead to depletion of Nrf2 (Yang et al., 2015). Moreover, disruption of Nrf2 signaling alters the angiogenic capacity of endothelial cells and expression of several anti-oxidant genes, leading to myocardial apoptosis and hypertrophy (Ke et al., 2013). Nrf2 accumulation in nuclei enhances expression of ARE-genes, thus reducing cardiac apoptosis (Kazakov et al., 2018). It was also discussed in a recently published review that NRF two is an important regulator on Mitochondrial Biogenesis, which play an important role in diabetic cardiomyopathy (Zheng et al., 2021). Similarly, it has been shown that activation of Nrf2 suppresses myocardial oxidative stress, apoptosis, fibrosis and hypertrophy in pressure overload induced by transverse aortic arch constriction (Wang et al., 2014).

Recent research has shown that Notoginsenoside R1 has a variety of pharmacological properties, including cardioprotective and neuroprotective, both *in vitro* and *in vivo* (Wang et al., 2008; Huang et al., 2016; Tabandeh et al., 2017). In this study, we focused on the cardioprotective effects of NGR1on high glucose-induced cell injury. We found that NGR1 attenuated cardiomyocyte hypertrophy was induced by high glucose concentration. Meanwhile, it was also shown that NGR1 protected H9c2 cardiomyocytes from high glucose-induced cell death and had promising anti-apoptotic effects. NGR1 treatment might be an optimal therapy for diabetic cardiomyopathy.

To investigate whether the AMPK pathway plays an important role in the reduction of high glucose-induced cardiomyocyte injury mediated by NGR1, we explored the effects of NGR1 and compound C on ANP and BNP expression and on high glucose-induced cell death and apoptosis. Compound C interfered with the cardioprotective effects of NGR1 by blocking AMPK activity, which suggests that AMPK is a key factor in the protective effect of NGR1 on glucose-induced cell injury. It has been shown that cardiomyocyte hypertrophy significantly increases in response to AMPK deficiency (Turdi et al., 2011), and AMPK is known to play a key role in the prevention of cardiomyocyte hypertrophy (Corbi et al., 2013). The results of the present study showed that NGR1 markedly reversed inhibition of Nrf2 and HO-1 expression induced by high glucose concentration. There is growing evidence that the Nrf2 pathway and expression of HO-1 are related to the anti-oxidant and anti-apoptotic effects of NGR1 (Zhang et al., 2019). When Nrf2 is activated, its downstream target HO-1 is transcribed (Khan et al., 2017). Studies have shown that upregulation of the Nrf2/HO-1 signaling pathway has a protective effect on inflammation, apoptosis, and fibrosis (Liao et al., 2017). Another study showed that ginsenoside Rg1 has anti-apoptotic activity, inhibiting cardiomyocyte hypertrophy by activating the Nrf2/HO-1 signaling pathway (Zhang et al., 2020). It was also found that compound C reduced Nrf2 and HO-1 levels, suggesting that AMPK upregulates Nrf2 and HO-1 expression and promotes Nrf2 and HO-1 protein synthesis and secretion. Several studies have demonstrated that AMPK activation increases Nrf2 and HO-1 signaling (Zimmermann et al., 2015; Joo et al., 2016). The present study demonstrated that Nrf2 knockdown resulted in the reversal of NGR1-mediated cardioprotection and was accompanied by markedly reduced HO-1 expression, consistent with expression of HO-1 being regulated by Nrf2. To confirm whether NGR1 activates Nrf2-mediated HO-1 signaling, Nrf2 was silenced in this study. HO-1 is thought to be a critical downstream effector of the Nrf2 signaling pathway, and the effect on HO-1 expression of transfection with Nrf2 siRNA supports this concept (Lee et al., 2012). Taken together, these results indicate that NGR1 activates Nrf2 signaling and increases HO-1 expression by activating the AMPK pathway.

In conclusion, our study provides novel insights into mechanisms underlying the cardioprotective effects of NGR1, clearly supporting the conclusion that treatment with NGR1 attenuates high glucose-induced cell injury via AMPK/Nrf2 signaling and its downstream target, the HO-1 pathway. The cardioprotective effects of NGR1 result from upregulation of AMPK/Nrf2 signaling and promotion of HO-1 expression in cardiomyocytes. Our findings suggest that NGR1 treatment might provide a novel therapy for diabetic cardiomyopathy. However, NGR1 may also play other roles in cardioprotection, and its specific mechanisms need to be further explored. Recently, secreted frizzled-related protein 2 and 5, a novel Adipokines, had showed to be protective in DCM and its effectiveness was mediated by the AMPK pathway (Huang and Huang, 2020; Wu J. et al., 2020, Wu et al., 2020 Y.). It is also reported that SFRP2 exerted cardioprotective effects by salvaging mitochondrial function in an AMPK-PGC1-α-dependent manner, which modulates mitochondrial dynamics and mitochondrial biogenesis, reducing oxidative stress and apoptosis (Ma et al., 2021). It would be interesting to explore whether NRG1 can play a protective role on DCM by increasing the expression of secreted frizzled-related proteins. Furthermore, in clinical treatment, traditional Chinese medicine primarily involves preparation of NGR1 as a monomer, and it may be possible to improve methods of extraction and purification. In future research, further efforts in the above areas will be required to provide a new direction for clinical treatment of diabetic cardiomyopathy.

## Data Availability

The raw data supporting the conclusions of this article will be made available by the authors, without undue reservation.
